# Familial Hypomagnesemia With Secondary Hypocalcemia: A Case Report

**DOI:** 10.7759/cureus.19847

**Published:** 2021-11-23

**Authors:** Nour Gazzaz, Maha Alghamdi

**Affiliations:** 1 Department of Pediatrics, King Abdulaziz University Faculty of Medicine, Jeddah, SAU

**Keywords:** familial hypomagnesemia, nonsense variant, secondary hypocalcemia, rare genetic disorder familial hypomagnesemia, refractory seizures

## Abstract

Familial hypomagnesemia with secondary hypocalcemia is a rare genetic disorder of magnesium metabolism that presents with refractory seizures during infancy. It is caused by loss-of-function mutations in the gene encoding transient receptor potential cation channel member 6 (TRPM6). Herein we report an infant who presented with refractory seizures that were brought under control by normalizing the magnesium level. Genetic analysis revealed a nonsense variant in the TRPM6 gene. Our case highlights the importance of evaluation for familial hypomagnesemia in any child with recurrent or refractory seizures.

## Introduction

Magnesium constitutes the majority of intracellular cations and is absorbed through the small and large intestine via two distinct transport pathways. It is also absorbed through the kidneys with the majority (60-70%) absorbed via the thick ascending limb of Henley (TAL) [1]. The balance of gastrointestinal absorption and renal excretion influences magnesium hemostasis. Magnesium is necessary for the function of numerous enzymes and the regulation of several ion channels, and the stabilization of negatively charged molecules [2].

Hypomagnesemia can occur for a variety of reasons, including insufficient intake of magnesium, abnormal gastrointestinal absorption, impaired renal conservation, or magnesium redistribution from extracellular to intracellular space. The Gitelman and Bartter syndromes are the most frequently occurring autosomal recessive conditions associated with hypomagnesemia. They are excluded if the blood gas, serum, and urine electrolytes showed no alkalosis, hypokalemia, hypocalciuria, and no hypermagnesuria. Familial hypomagnesemia with hypercalciuria and nephrocalcinosis is an autosomal recessive condition caused by a mutation in the CLDN16 gene [3]. It is associated with signs of renal magnesium deficiency, elevated serum calcium levels, and nephrocalcinosis or nephrolithiasis on renal ultrasound. Additionally, certain medications, such as aminoglycosides, digoxin, diuretics, proton pump inhibitors, and osmotic agents, can cause hypomagnesemia [4].

Familial hypomagnesemia with secondary hypocalcemia (FHSH) is a rare autosomal recessive disorder (OMIM# 602014) that typically manifests in early childhood and is characterized by abnormally low serum magnesium levels [5,6]. Decreased magnesium causes impaired magnesium-dependent adenyl cyclase generation of cyclic adenosine monophosphate (cAMP), which decreases the release of parathyroid hormone (PTH) and in turn, calcium levels are decreased [7]. FHSH is caused by mutations in the transient receptor potential cation channel member (TRPM6) gene that codes for the TRPM6 protein. This protein is a member of the melastatin family of transient receptor potential (TRP) cation channels and functions as an intermediary for a magnesium permeable channel found in the brush-border membrane of the small intestine and apical membrane of magnesium-reabsorbing tubules in the kidney [8]. Most of the mutations identified in FHSH patients result in the full loss of TRPM6 function which results in diminished intestinal magnesium reabsorption and renal wasting [9].

The disease typically manifests during the first months of life by increased neuromuscular excitability, such as muscle spasms or tetany and refractory seizure [5]. Without treatment, there is an increased risk of developmental delay, severe brain damage, severe cardiomyopathy, and death [10]. Management should be initiated immediately by administering intravenous magnesium, and when stable this should be followed by high doses of oral magnesium lifelong to alleviate clinical symptoms and aid in the restoration of calcium metabolism [9].

Our case underlines the importance of pediatricians expanding their knowledge of this uncommon diagnosis. Despite its rarity, FHSH is a treatable disease. 

## Case presentation

A seven-month-old baby girl from Myanmar who is the second child of consanguineous parents presented to our hospital with recurrent generalized tonic-clonic seizures since the age of three months.

The first seizure was reported to be fever-induced at the age of three months. At the time, she was treated at another center as a case of meningitis. The diagnosis of epilepsy was made based on the recurrent generalized tonic-clonic seizures for which she was started on levetiracetam and phenobarbitone. She was also diagnosed with rickets because of the low calcium levels and was treated with calcium, vitamin D, and was also prescribed oral magnesium for low magnesium. She had good medication compliance and was seizure-free until the age of seven months when she presented to our hospital with three days history of a generalized tonic-clonic seizure. She had an increased frequency - six to seven times a day lasting approximately seven minutes despite being on an appropriate dose of antiepileptics. The only remarkable history was that the family ran out of magnesium supplements for five days. There was no fever or other infection-related symptoms in the prior days. She was born at full term following an uncomplicated pregnancy with no evidence of polyhydramnios and weighing 3500 g at birth. Her prenatal and postnatal history was normal. She had gross motor development delay (she could sit with assistance but could not reach for objects); her overall developmental age was approximately four to five months. Her family history was unremarkable; she had no family history of epilepsy or neurological abnormalities and was free of known parathyroid, thyroid, or renal disease.

Physical examination revealed normal growth parameters: weight is 7.5 kg (50th centile), length is 68 cm (75th centile), and head circumference is 43 cm (50th centile).

There were no dysmorphic features and no neurocutaneous or meningeal abnormalities. Neurological examination was unremarkable at the time of presentation, with normal tone and reflexes and spontaneous movement of all four limbs.

The laboratory workup showed severely low magnesium and mildly decreased calcium levels. She had no evidence of parathyroid abnormality and both calcium to creatinine ratio and magnesium excretion were normal (Table 1). Stool analysis was not done. Ultrasound examinations of the abdomen and pelvis revealed no signs of nephrocalcinosis. Electroencephalogram (EEG) showed no epileptiform discharges and brain MRI was unremarkable.

**Table 1:** The laboratory investigations

Test	Patient’s Result	Reference Intervals
Magnesium	0.23 mmol/L	0.70 – 1.0
Calcium	2.10 mmol/L	2.12 – 2.52
Phosphorus	2.39 mmol/L	0.81 – 1.58
Parathyroid hormone	3.4 pmol/l	1.18 – 8.43
Alkaline phosphatase	266 U/L	60 – 306
Sodium	137 mmol/L	135 – 144
Potassium	4.8 mmol/L	3.3 – 5.1
Albumin	42 g/L	40 – 50
Urea	2.9 mmol/L	5 – 25
Creatinine	25 μmol/L	53 – 115
Urinary calcium/creatinine ratio	Normal	
The fraction excretion of magnesium	2%	< 4%

In the emergency department, the levetiracetam and phenobarbitone doses were increased. Given the severe hypomagnesemia and mild hypocalcemia, the patient was given two boluses of intravenous magnesium sulfate at 50 mg/kg and intravenous calcium gluconate infusion at 30 mg/kg/dose. After which the serum magnesium increased and the calcium levels normalized. She was hospitalized in the pediatric unit to investigate the underlying causes of the refractory seizure. The clinical and laboratory findings suggested that the electrolyte imbalance was the most likely cause for her presentation. Numerous pediatric subspecialties were consulted to investigate the underlying cause of hypomagnesemia and secondary hypocalcemia. The endocrine team ruled out hypoparathyroidism as a cause of the low calcium as her PTH was normal. The nephrology team ruled out renal magnesium loss because the initial fraction excretion of magnesium was normal. Additionally, intestinal loss was determined to be an unlikely cause of hypomagnesemia as there was no history of laxative use and no signs or symptoms of malabsorption. At this point, the diagnosis of FHSH as the cause of magnesium homeostasis disruption was suspected. Singleton whole-exome sequencing was sent for confirmation and revealed a pathogenic homozygous nonsense variant in TRMP6 [NM_017662.5: c.608G>A (p.Trp203*)]. This null variant leads to loss of protein function which is consistent with the disease mechanism. It is located at the conserved N terminus and is absent in gnomAD [11]. Lastly, it is predicted by the in silico tools to be deleterious.

After 48 hours, the antiepileptic medications and calcium gluconate were discontinued, and the calcium level remained normal at 2.41 mmol/L and she was switched to high doses of oral magnesium supplements. At follow-up, two months after her discharge, the magnesium level stayed within the normal range of 0.68 mmol/L, and she continued to be seizure-free.

## Discussion

We report a case of FHSH in an infant who presented with refractory seizures in early infancy. FHSH is a rare autosomal recessive disorder caused by transient receptor potential melastatin 6 (TRPM6) gene mutations which result in a defect in the magnesium permeable ion channel. Only about 100 cases have been reported in the literature to date, with both sexes equally affected [12]. TRPM6 is expressed on the intestine and the apical membrane of the kidney's distal convoluted tubules (DCT) [4,13]. The pathophysiology is related to impaired intestinal and renal reabsorption of magnesium, resulting in serum hypomagnesemia [14]. It is thought that affected individuals have an isolated defect of intestinal magnesium absorption with normal or low renal magnesium excretion at diagnosis as seen in our case; the renal magnesium leak becomes apparent only when the serum magnesium concentration increases, indicating a decreased renal magnesium threshold [15]. FHSH typically presents in the first months of life with convulsions, muscle spasms, or tetany. Intravenous or intramuscular magnesium is administered to alleviate symptoms, followed by lifelong treatment with high-dose oral magnesium [15]. Despite the fact that patients with the TRPM6 mutation have impaired intestinal magnesium absorption, they respond to high doses of oral magnesium. This is due to the intestine's dual magnesium transport pathways [7]. The first is a transcellular active transporter that is deficient in FHSH (TRPM6); it is saturable at high luminal magnesium concentrations, which is important for function at low luminal magnesium concentrations. The second mechanism is a passive paracellular transport that is proportional to the amount of magnesium in the intraluminal space. As a result, serum magnesium levels improve but remain within the subnormal range (0.5-0.6 mmol/L) [7]. 

Our patient presented with refractory seizure following discontinuation of magnesium supplement. Magnesium deficiency impairs the magnesium-dependent adenyl cyclase's production of cyclic adenosine monophosphate (cAMP), which reduces parathyroid hormone (PTH) release and, consequently, calcium levels [7]. Our patient had very mild hypocalcemia that was corrected with magnesium replacement. She has been monitored on a regular basis, with serial serum magnesium measurements and dose adjustments of oral magnesium made as needed. The magnesium level remained at the subnormal level and the oral dose is adjusted according to her weight. She has been catching up in her development and at her most recent check-up at the age of nine months, her development was up to date, with no evidence of neurological sequala. 

Our case underlines the importance of pediatricians expanding their knowledge of this uncommon diagnosis. FHSH is a treatable disease, early detection and treatment can prevent devastating neurological consequences. Any child who has seizures along with hypomagnesemia and hypocalcemia should be evaluated for the possibility of FHSH. Genetic counseling should be offered to the family for prenatal counseling, early detection, and management. 

## Conclusions

FHSH should be investigated in an infant presenting with refractory or recurrent seizures, particularly when serum magnesium levels are as low as 0.2 mmol/L. Genetic testing is critical because it aids in diagnosis and counseling and contributes to our understanding of magnesium homeostasis.
